# Supplementary material for: Geometry and evolution of the ecological niche in plant-associated microbes
Source: Nat Commun. 2020 Jun 11;11:2955. doi: 10.1038/s41467-020-16778-5 (PMC7289842; doi:10.1038/s41467-020-16778-5)
Supplement: Supplementary file 1 — Supplementary Information [file 41467_2020_16778_MOESM1_ESM.pdf]

Supplementary Information PDF

Geometry and evolution of the ecological niche in plant-associated microbes

Authors: Thomas M. Chaloner[1], Sarah J. Gurr[1,2] & Daniel P. Bebber[1]*.
Affiliations:
[1]Department of Biosciences, University of Exeter, Exeter EX4 4QJ, UK.
[2]Department of Biosciences, Utrecht University, Paduallaan 8, Netherlands.
*Correspondence to: d.bebber@exeter.ac.uk

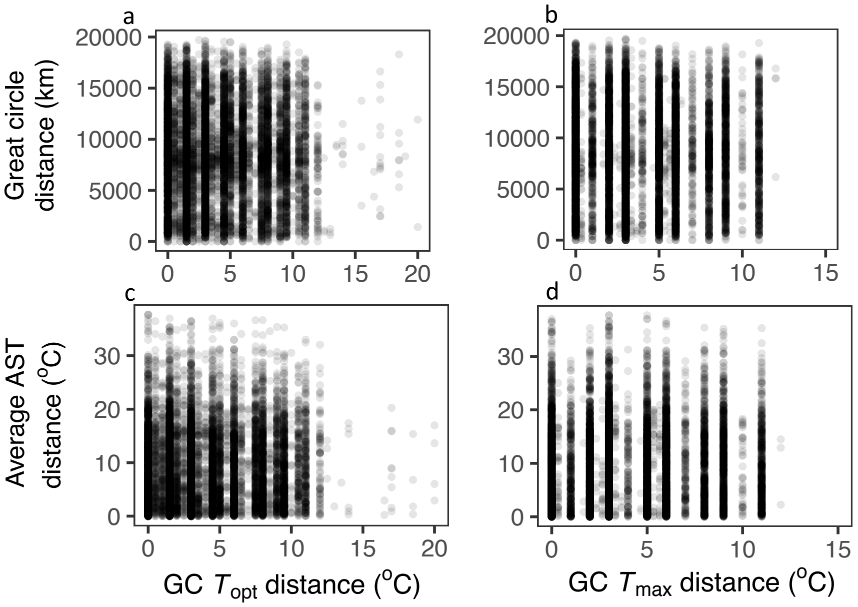

Supplementary Fig. 1. Analysis of spatial correlation on phylogenetic signals calculated for *Phytophthora* species cardinal temperature.
(a, c) GC T_{opt}. (b, d) GC T_{max}. (a, b) Relationship between GC temperature response distance (°C) and great circle distance (km). (a) Mantel correlation (MC) = -0.016 (95% CI -0.021 - -0.012), p = 0.013. (b) MC = 0.013 (0.009 - 0.018), p = 0.016. (c, d) Relationship between GC temperature response distance (°C) and average air surface temperature (AST) (°C). (c) MC = 0.020 (0.014 - 0.026), p = 0.009. (d) MC = 0.044 (0.037 - 0.051), p = 0.001. $N_{species}$ = 31; $N_{distances}$ = 1,367,031. Figures show 10,000 randomly sampled data points. All Mantel correlations are near zero. Hence, we ignore the influence of spatial effects on our analysis of cardinal temperature phylogenetic signal.

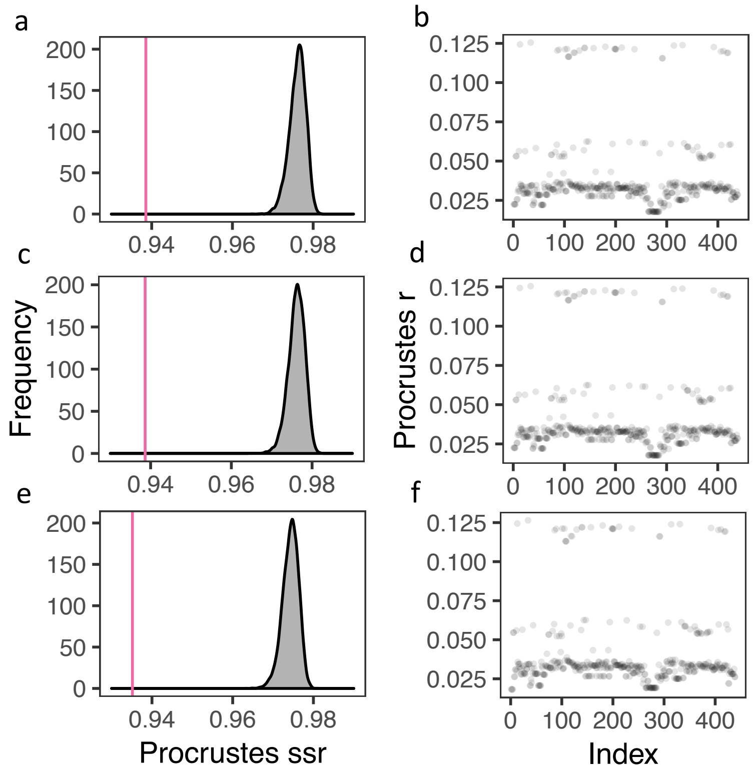

Supplementary Fig. 2. Host/*Phytophthora* co-phylogenetic association analysis. (a, c, e) The observed best-fit (pink lines) Procrustean super-imposition (ssr) (0.939, 0.939, 0.935) were better (lower) than the same for any of the ensemble of network randomisations in each null model (grey distribution) (*N* = 10,000). (b, d, f) Procrustes residuals (r) for all interactions in each host-pathogen network (*N* = 439). (a, b) Bayesian *Phytophthora* species phylogeny. (c, d) Maximum likelihood *Phytophthora* species phylogeny. (e, f) Maximum parsimony *Phytophthora* species phylogeny. All *Phytophthora* species phylogenies were extracted from ref.[1] (TreeBASE S19303).

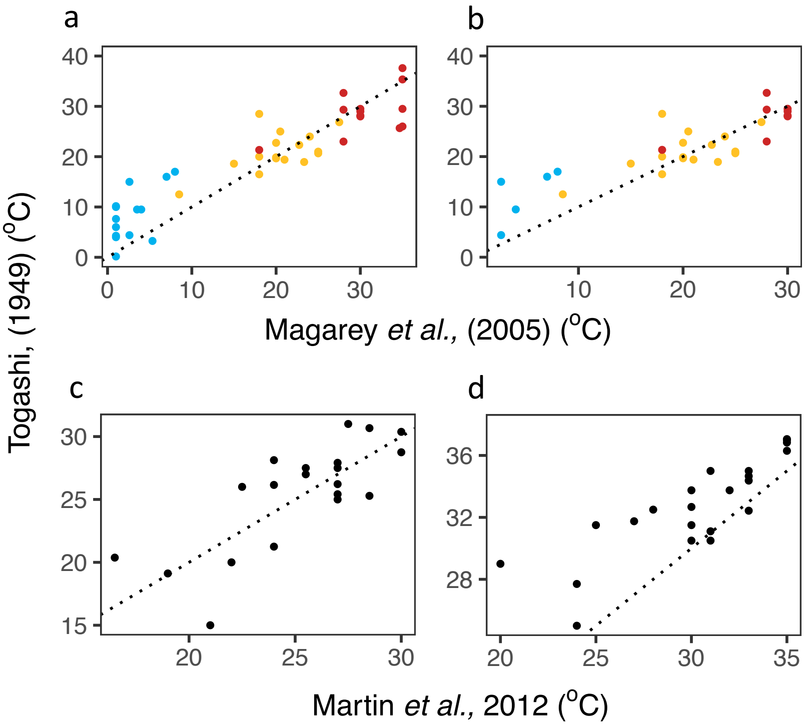

Supplementary Fig. 3. Relationship between species CT estimates.
(a, b) Relationship between IN cardinal temperature from ref. [2] and ref. [3]. Colour refers to estimates of T_{min} (blue), T_{opt} (yellow) and T_{max} (red). (a) Inclusion of all available data, root mean square error (RMSE) = 5.15 °C, N = 43. (b) Exclusion of data where T_{min} and T_{max} were respectively recorded as 1 °C and 35 °C in ref. [2], RMSE = 4.73 °C, N = 29. Relationship between GC (c) T_{opt} and (d) T_{max} estimates from ref. [2] and ref. [4]. (c) RMSE = 2.65 °C, N = 20. (d) RMSE = 3.34 °C, N = 22. Dotted line indicates identity relationship.

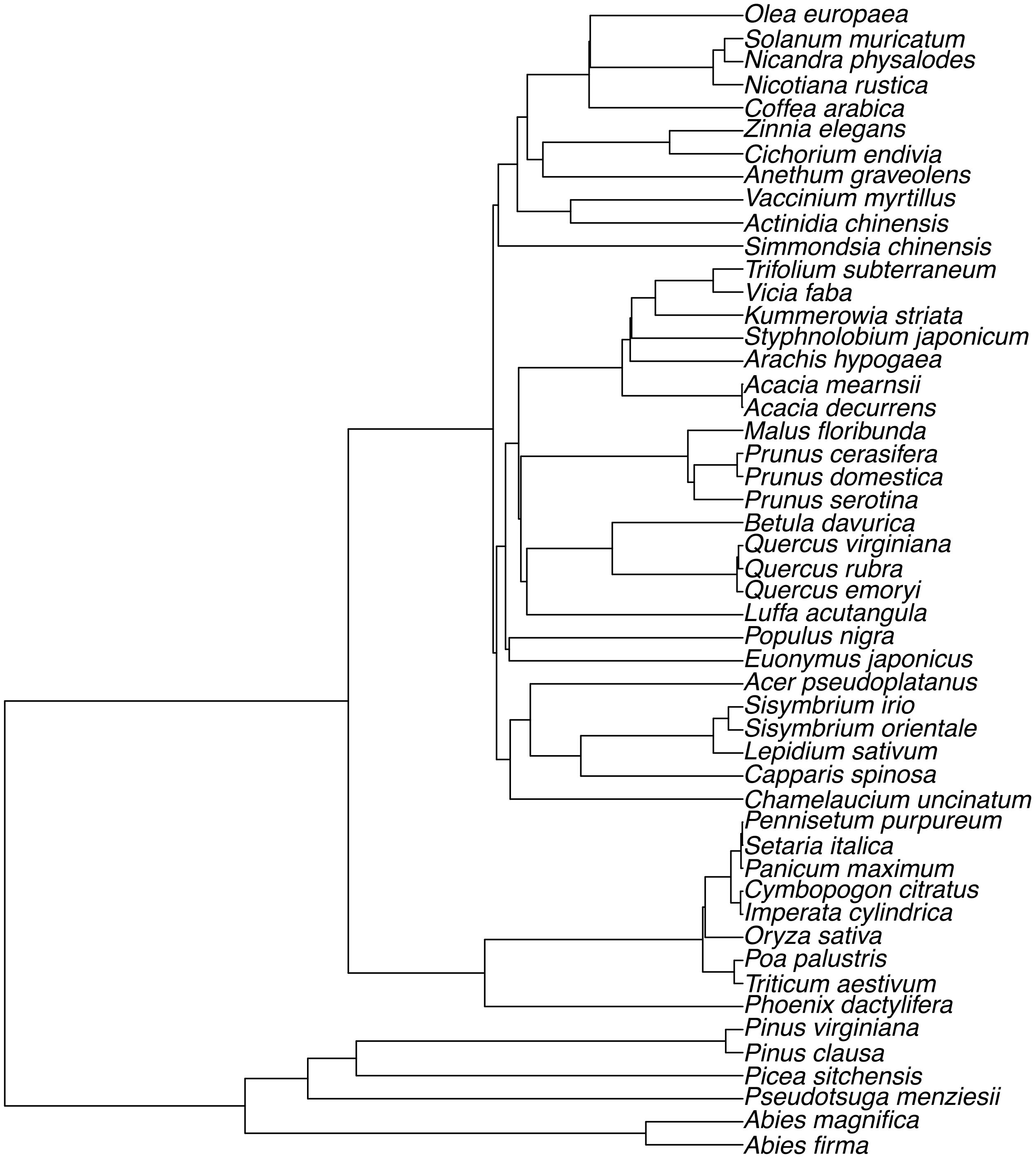

Supplementary Fig. 4. Unprocessed host phylogeny constructed using S. PhyloMaker. For clarity, 50 tips have been randomly extracted for presentation. Hosts recorded no further than species. Hosts not recorded as far as species in the Plantwise database were excluded. Hosts not identifiable at genus-level in S.PhyloMaker are excluded.

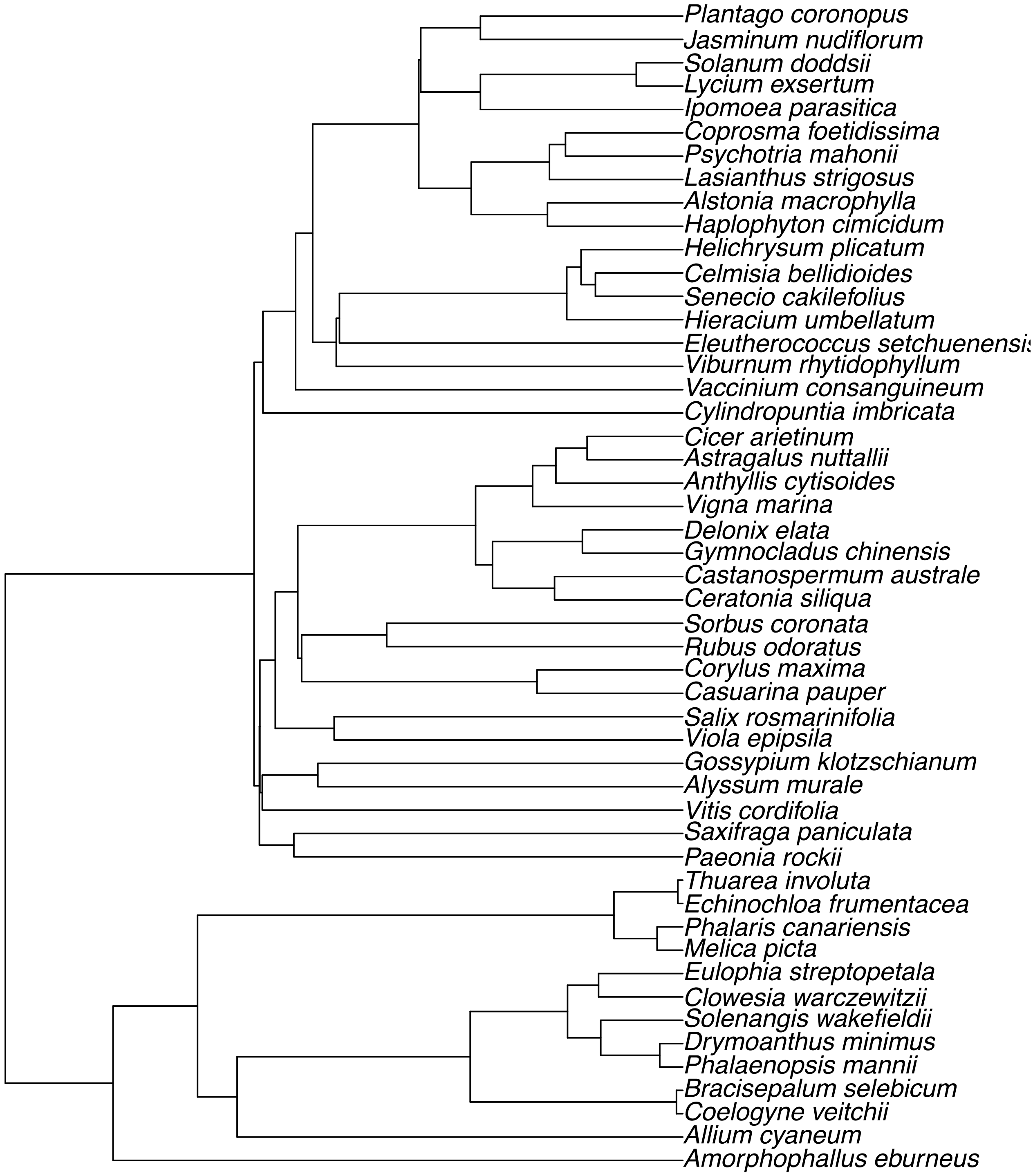

Supplementary Fig. 5. Processed host phylogeny constructed using S. PhyloMaker.
For clarity, 50 tips have been randomly extracted for presentation. Hosts recorded no further than species. Hosts not identifiable at genus-level in S.PhyloMaker were excluded.

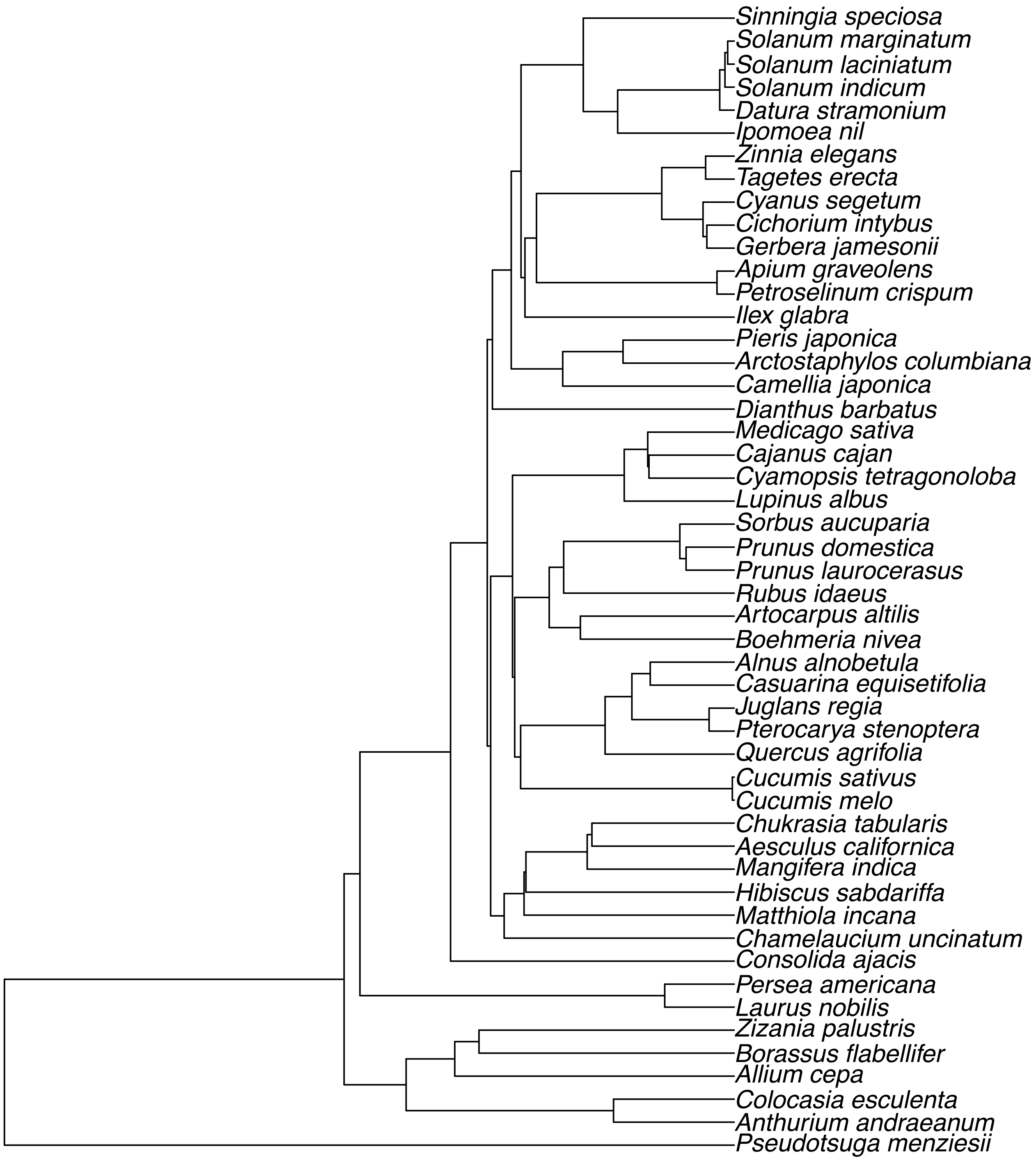

Supplementary Fig. 6. *Phytophthora* spp. host phylogeny constructed using S. PhyloMaker. For clarity, 50 tips have been randomly extracted for presentation. Hosts recorded no further than species. Hosts not identifiable at genus-level in S.PhyloMaker were excluded.

Supplementary Table 1. Summary of temperature responses of fungi and oomycetes for different processes. Medians with interquartile ranges in parentheses. Sample sizes shown (*N*). Note that *F. oxysporum formae speciales* are included in these calculations.

Fungi					
Process	T_{min} (°C)	T_{opt} (°C)	T_{max} (°C)	T_{range} (°C)	Skew
Disease development	9.8 (3.7 – 14.0)<br>*N* = 89	23.5 (19.9 – 26.6)<br>*N* = 107	30.7 (27.2 – 33.1)<br>*N* = 71	20.0 (17.3 – 25.6)<br>*N* = 64	0.60 (0.51 – 0.73)<br>*N* = 58
Fructification	10.0 (6.9 – 15.0)<br>*N* = 31	22.9 (18.4 – 28.0)<br>*N* = 36	30.0 (27.5 – 33.0)<br>*N* = 33	19.2 (15.6 – 24.6)<br>*N* = 30	0.60 (0.49 – 0.71)<br>*N* = 22
Growth in culture	6.0 (4.0 – 9.4)<br>*N* = 425	25.4 (22.8 – 28.0)<br>*N* = 502	35.0 (32.5 – 37.5)<br>*N* = 467	28.0 (26.0 – 30.8)<br>*N* = 400	0.68 (0.62 – 0.75)<br>*N* = 370
Infection	10.0 (7.0 – 15.1)<br>*N* = 76	21.1 (18.0 – 26.1)<br>*N* = 98	30.0 (26.2 – 33.4)<br>*N* = 77	19.6 (15.6 – 24.8)<br>*N* = 62	0.58 (0.48 – 0.72)<br>*N* = 52
Spore germination	6.0 (3.0 – 8.1)<br>*N* = 143	23.0 (19.0 – 26.2)<br>*N* = 180	32.4 (30.0 – 36.0)<br>*N* = 150	27.0 (23.5 – 30.0)<br>*N* = 133	0.62 (0.51 – 0.69)<br>*N* = 126
Sporulation	11.8 (5.1 – 15.0)<br>*N* = 38	24.0 (19.2 – 28.4)<br>*N* = 46	31.7 (26.4 – 34.4)<br>*N* = 38	20.5 (16.8 – 25.2)<br>*N* = 36	0.62 (0.52 – 0.68)<br>*N* = 30

Oomycetes					
Process	T_{min} (°C)	T_{opt} (°C)	T_{max} (°C)	T_{range} (°C)	Skew
Disease development	9.6 (6.8 – 11.2)<br>*N* = 12	22.5 (20.2 – 28.0)<br>*N* = 13	30.0 (29.2 – 32.5)<br>*N* = 11	20.5 (20.0 – 22.6)<br>*N* = 11	0.63 (0.58 – 0.68)<br>*N* = 10
Fructification	10.0 (3.0 – 16.7)<br>*N* = 5	21.2 (17.2 – 24.2)<br>*N* = 6	26.5 (22.4 – 32.5)<br>*N* = 7	15.0 (13.3 – 21.8)<br>*N* = 5	0.81 (0.76 – 0.82)<br>*N* = 4
Growth in culture	7.7 (6.3 – 9.0)<br>*N* = 34	27.2 (25.9 – 30.0)<br>*N* = 40	35.0 (32.5 – 37.0)<br>*N* = 39	27.0 (25.5 – 30.0)<br>*N* = 33	0.75 (0.67 – 0.79)<br>*N* = 30
Infection	10.1 (8.1 – 14.2)<br>*N* = 14	19.5 (16.3 – 22.7)<br>*N* = 14	31.5 (29.5 – 34.0)<br>*N* = 15	21.7 (18.8 – 25.0)<br>*N* = 13	0.56 (0.51 – 0.64)<br>*N* = 10
Spore germination	6.5 (4.0 – 9.3)<br>*N* = 19	19.0 (16.4 – 23.7)<br>*N* = 27	28.8 (26.4 – 31.6)<br>*N* = 23	22.6 (19.2 – 24.4)<br>*N* = 19	0.58 (0.49 – 0.66)<br>*N* = 18
Sporulation	7.4 (5.5 - 8.6)<br>*N* = 7	19.1 (16.2 – 19.8)<br>*N* = 7	28.8 (23.9 – 29.5)<br>*N* = 7	20.8 (19.8 – 21.2)<br>*N* = 7	0.53 (0.49 - 0.55)<br>*N* = 6

Supplementary Table 2. Difference between temperature responses for growth in culture (GC) and other processes within species. Positive values indicate that the alternative process has a higher value than GC. Fungi and oomycetes are not differentiated because only a small number of pairwise comparisons were available for oomycetes. Cardinal temperatures were compared using two-sided *t*-tests due to large differences in sample size. The Holm-Bonferroni adjusted significance level was 0.013 (i.e. p-values below 0.013 indicate the null hypothesis can be rejected).

Response	Process	Difference (°C)	95% CI	*N*	df	*t*	*p*
T_{min}	Disease development	2.5	1.46, 3.51	77	76	4.82	7.1e-06
	Fructification	4.7	3.08, 6.28	30	29	5.98	1.7e-06
	Infection	3.0	1.82, 4.27	54	53	4.98	7.1e-06
	Spore germination	0.3	-0.55, 1.21	88	87	0.75	0.46
	Sporulation	3.1	1.93, 4.25	38	37	5.40	4.1e-06
T_{opt}	Disease development	-2.2	-3.02, -1.31	91	90	-5.03	2.5e-06
	Fructification	-1.4	-3.32, 0.48	35	34	-1.51	0.14
	Infection	-3.1	-4.22, -1.97	80	79	-5.46	5.3e-07
	Spore germination	-0.2	-0.84, 0.39	120	119	-0.72	0.48
	Sporulation	-2.1	-3.47, -0.82	40	39	-3.28	0.0022
T_{max}	Disease development	-4.1	-5.38, -2.87	61	60	-6.56	1.4e-08
	Fructification	-4.1	-6.19, -2.02	33	32	-4.02	0.00034
	Infection	-3.8	-5.13, -2.54	58	57	-5.92	1.9e-07
	Spore germination	0.1	-0.48, 0.74	94	93	0.43	0.67
	Sporulation	-3.0	-4.47, -1.54	35	34	-4.16	2e-04
T_{range}	Disease development	-6.2	-7.99, -4.51	56	55	-7.20	1.8e-09
	Fructification	-8.7	-11.61, -5.82	28	27	-6.17	1.3e-06
	Infection	-6.3	-8.22, -4.38	44	43	-6.61	4.7e-08
	Spore germination	-0.2	-1.44, 1.05	84	83	-0.31	0.76
	Sporulation	-5.9	-7.73, -4.02	35	34	-6.43	2.4e-07
Skew	Disease development	-0.04	-0.08, 0.01	51	50	-1.64	0.11
	Fructification	-0.04	-0.13, 0.05	21	20	-1.00	0.33
	Infection	-0.04	-0.10, 0.01	41	40	-1.54	0.13
	Spore germination	0.00	-0.03, 0.02	79	78	-0.29	0.77
	Sporulation	-0.08	-0.14, -0.02	29	28	-2.67	0.013

Supplementary Table 3. Correlation coefficients and RMSE (°C) among T_{opt} values for different processes within species. 95% confidence intervals are given in parentheses. Degrees of freedom (df) and *t*-test statistics vs. zero correlation are given. *P*-values are not given due to multiplicity of tests. NA denotes insufficient data. Pearson correlations were two-sided.

Correlation coefficients					
T_{opt}	Disease development	Fructification	Growth in culture	Infection	Spore germination
Disease development	-	-	-	-	-
Fructification	0.69 (0.25 – 0.89) df = 12, t = 3.28	-	-	-	-
Growth in culture	0.65 (0.51 – 0.75) df = 89, t = 7.99	0.44 (0.13 - 0.68) df = 33, t = 2.83	-	-	-
Infection	0.91 (0.85 - 0.94) df = 66, t = 17.61	0.77 (0.45 - 0.92) df = 14, t = 4.57	0.64 (0.49 - 0.75) df = 78, t = 7.38	-	-
Spore germination	0.72 (0.56 - 0.83) df = 52, t = 7.41	0.35 (-0.13 - 0.69) df = 17, t = 1.52	0.66 (0.54 - 0.75) df = 118, t = 9.52	0.78 (0.65 - 0.86) df = 58, t = 9.44	-
Sporulation	0.72 (0.42 - 0.88) df = 19, t = 4.52	NA	0.71 (0.52 - 0.84) df = 38, t = 6.28	0.81 (0.60 - 0.92) df = 21, t = 6.40	0.78 (0.59 - 0.89) df = 28, t = 6.67

RMSE					
T_{opt}	Disease development	Fructification	Growth in culture	Infection	Spore germination
Disease development	-	-	-	-	-
Fructification	4.53	-	-	-	-
Growth in culture	4.62	5.63	-	-	-
Infection	2.40	5.31	5.91	-	-
Spore germination	4.25	6.00	3.39	3.92	-
Sporulation	4.47	NA	4.62	3.76	4.93

Supplementary Table 4. Correlation coefficients among T_{range} values for different processes within species. 95% confidence intervals are given in parentheses. Degrees of freedom (df) and *t*-test statistics vs. zero correlation are given. *P*-values are not given due to multiplicity of tests. NA denotes insufficient data. Pearson correlations were two-sided.

Correlation coefficients					
T_{opt}	Disease development	Fructification	Growth in culture	Infection	Spore germination
Disease development	-	-	-	-	-
Fructification	0.46 (-0.24 - 0.84) df = 8, *t* = 1.45	-	-	-	-
Growth in culture	0.24 (-0.03 - 0.47) df = 54, *t* = 1.80	-0.05 (-0.42 - 0.33) df = 26, *t* = -0.26	-	-	-
Infection	0.91 (0.85 - 0.95) df = 44, *t* = 14.82	0.34 (-0.36 - 0.80) df = 8, *t* = 1.04	0.27 (-0.03 - 0.52) df = 42, *t* = 1.80	-	-
Spore germination	0.39 (0.06 - 0.64) df = 32, *t* = 2.39	0.25 (-0.32 – 0.69) df = 12, *t* = 0.91	0.40 (0.21 - 0.57) df = 82, *t* = 3.99	0.29 (-0.03 - 0.55) df = 37, *t* = 1.83	-
Sporulation	0.46 (-0.05 - 0.78) df = 14, *t* = 1.94	NA	0.57 (0.29 - 0.76) df = 33, *t* = 3.96	0.29 (-0.22 - 0.68) df = 15, *t* = 1.18	0.38 (-0.04 - 0.68) df = 21, *t* = 1.87

Supplementary Table 5. Correlation between abiotic niche breadth and biotic niche breadth. Abiotic niche breadth was estimated as T_{range} or $T_{range0.5}$, and biotic niche breadth as log_{10+1}-transformed host phylogenetic diversity calculated from either the processed or unprocessed host phylogeny. Parameter estimate, 95% confidence intervals, and *t*-test statistics vs. zero correlation are given. Pearson correlations were two-sided. The Holm-Bonferroni adjusted significance level was < 0.013, i.e. no null hypotheses of Cor = 0 were rejected.

	T_{range} / processed host phylogeny				
Process	Cor	95% CI	df	*t*	*p*
Disease development	0.203	-0.056, 0.436	57	1.56	0.12
Fructification	0.039	-0.389, 0.453	20	0.17	0.86
Growth in culture	0.139	0.004, 0.270	207	2.02	0.044
Infection	-0.011	-0.270, 0.251	55	-0.08	0.94
Spore germination	0.208	0.017, 0.385	103	2.16	0.033
Sporulation	-0.048	-0.435, 0.354	23	-0.23	0.82

	T_{range} / unprocessed host phylogeny				
Process	Cor	95% CI	df	*t*	*p*
Disease development	0.247	-0.010, 0.473	57	1.93	0.059
Fructification	-0.081	-0.486, 0.353	20	-0.36	0.72
Growth in culture	0.174	0.038, 0.304	202	2.51	0.013
Infection	0.038	-0.225, 0.296	55	0.28	0.78
Spore germination	0.212	0.021, 0.389	102	2.20	0.03
Sporulation	-0.087	-0.466, 0.319	23	-0.42	0.68

	$T_{range0.5}$ / processed host phylogeny				
Process	Cor	95% CI	df	*t*	*p*
Disease development	0.133	-0.142, 0.389	51	0.96	0.34
Fructification	-0.259	-0.658, 0.253	15	-1.04	0.32
Growth in culture	0.027	-0.112, 0.165	198	0.38	0.70
Infection	0.015	-0.265, 0.292	48	0.10	0.92
Spore germination	0.053	-0.146, 0.248	97	0.53	0.60
Sporulation	0.173	-0.268, 0.554	20	0.78	0.44

	$T_{range0.5}$ / unprocessed host phylogeny				
Process	Cor	95% CI	df	*t*	*p*
Disease development	0.188	-0.087, 0.436	51	1.36	0.18
Fructification	-0.264	-0.661, 0.249	15	-1.06	0.31
Growth in culture	0.024	-0.117, 0.164	193	0.33	0.74
Infection	-0.011	-0.289, 0.268	48	-0.08	0.94
Spore germination	0.056	-0.144, 0.252	96	0.55	0.58
Sporulation	0.302	-0.137, 0.642	20	1.41	0.17

Supplementary Table 6. Difference between temperature responses for growth in culture (GC) and other processes within species with ambiguous species records excluded. Medians with interquartile ranges (IQR) and sample size (*N*). Positive values indicate that the alternative process has a higher value than GC. Fungi and oomycetes are not differentiated because only a small number of pairwise comparisons were available for oomycetes.

Response	Process	Median Difference (°C)	IQR	*N*
T_{min}	Disease development	2.0	0.00, 5.05	61
	Fructification	3.4	1.50, 6.56	24
	Infection	2.5	0.00, 7.00	41
	Spore germination	0.0	-2.05, 2.60	72
	Sporulation	3.8	0.89, 6.00	31
T_{opt}	Disease development	-1.0	-3.87, 0.00	70
	Fructification	0.0	-3.06, 0.71	27
	Infection	-2.0	-5.28, 0.00	61
	Spore germination	-0.2	-1.69, 1.03	95
	Sporulation	-1.2	-5.27, 0.00	34
T_{max}	Disease development	-3.4	-5.25, -0.74	48
	Fructification	-2.4	-6.00, -0.50	27
	Infection	-3.3	-5.16, -0.15	45
	Spore germination	0.3	-1.14, 2.00	75
	Sporulation	-3.0	-5.50, -1.00	29
T_{range}	Disease development	-6.0	-11.00, -1.97	44
	Fructification	-6.1	-12.67, -2.56	22
	Infection	-5.8	-10.30, -1.86	34
	Spore germination	0.0	-3.56, 2.87	68
	Sporulation	-7.0	-9.50, -3.97	29
Skew	Disease development	-0.03	-0.14, 0.09	39
	Fructification	-0.07	-0.10, 0.03	15
	Infection	-0.03	-0.15, 0.08	32
	Spore germination	0.00	-0.11, 0.07	63
	Sporulation	-0.09	-0.15, 0.04	25

Supplementary Table 7. Influence of possible data reporting uncertainties on within species differences between T_{opt} and T_{range} for growth in culture (GC) and other processes. Positive values indicate that the alternative process has a higher value than GC. Fungi and oomycetes are not differentiated because only a small number of pairwise comparisons were available for oomycetes. Cardinal temperatures were compared using two-sided *t*-tests due to large differences in sample size. The Holm-Bonferroni adjusted significance level was < 0.14, (i.e. p-values below 0.14 indicate the null hypothesis can be rejected).

Response	Process	Difference (°C)	95% CI	df	*t*	*p*
T_{opt}	Disease development	-2.1	-3.00, -1.27	90	-4.90	4.1e-06
	Fructification	-1.4	-3.32, 0.48	34	-1.52	0.14
	Infection	-3.0	-4.10, -1.82	79	-5.17	1.7e-06
	Spore germination	-0.20	-0.82, 0.42	119	-0.65	0.52
	Sporulation	-2.1	-3.47, -0.82	39	-3.28	0.0022
T_{range}	Disease development	-6.2	-7.91, -4.47	55	-7.22	1.6e-09
	Fructification	-9.0	-12.19, -5.91	27	-5.92	2.6e-06
	Infection	-6.1	-7.91, -4.21	43	-6.60	4.9e-08
	Spore germination	0.60	-0.69, 1.87	83	0.91	0.36
	Sporulation	-6.2	-8.02, -4.47	34	-7.15	2.9e-08

Supplementary Table 8. Correlation between abiotic niche breadth and biotic niche breadth with ambiguous species records excluded. Abiotic niche breadth was estimated as T_{range} and biotic niche breadth as $\log_{10+1}$-transformed host phylogenetic diversity calculated from the processed host phylogeny. Parameter estimate and 95% confidence intervals are given. Pearson correlations were two-sided.

	T_{range} / processed host phylogeny		
Process	Cor	95% CI	df
Disease development	0.202	-0.087, 0.460	46
Fructification	0.024	-0.448, 0.486	16
Growth in culture	0.145	-0.003, 0.287	173
Infection	-0.101	-0.377, 0.192	45
Spore germination	0.262	0.063, 0.441	92
Sporulation	-0.262	-0.616, 0.180	20

Supplementary Table 9. Summary information for analysis of niche co-specialisation. Note that host number refers to all pathogens identified in both the Togashi dataset and the Plantwise database (excluding hosts discussed in Methods section). However, pathogens included in analyses of niche co-specialisation (Pathogens identified) required at least one T_{range} or $T_{range0.5}$ for at least one biological process in the Togashi dataset.

Phylogeny	Hosts in phylogeny	Hosts identified to species	Hosts identified to genus	Temperature range	Pathogens identified
Unprocessed	1016	914	102	T_{range}	259
Unprocessed	1016	914	102	$T_{range0.5}$	246
Processed	15,982	15,880	102	T_{range}	264
Processed	15,982	15,880	102	$T_{range0.5}$	251

Supplementary Table 10. Influence of data reporting uncertainties on correlation between abiotic niche breadth and biotic niche breadth. Abiotic niche breadth was estimated as T_{range} and biotic niche breadth as $\log_{10+1}$-transformed host phylogenetic diversity calculated from the processed host phylogeny. Parameter estimate, 95% confidence intervals, and *t*-test statistics vs. zero correlation are given. Pearson correlations were two-sided.

T_{range} / processed host phylogeny					
Process	Cor	95% CI	df	*t*	*p*
Disease development	0.181	-0.079, 0.418	57	1.39	0.17
Fructification	0.040	-0.388, 0.454	20	0.18	0.86
Growth in culture	0.120	-0.016, 0.252	207	1.74	0.084
Infection	0.006	-0.255, 0.266	55	0.04	0.97
Spore germination	0.165	-0.028, 0.346	103	1.70	0.093
Sporulation	0.009	-0.388, 0.403	23	0.04	0.97

Supplementary Table 11. Pathogen species names updated in the Plantwise database to improve matching to the Togashi database. Pathogen species names were updated according to either the IF/SF or Mycobank databases. A pathogens name was only updated in the Plantwise database if the updated current name was present in the Togashi dataset. Authors of all updated species names above matched those in the Togashi dataset. Note that whilst all pathogens listed above were present in both the Togashi dataset and the Plantwise database, only those with T_{range} or $T_{range0.5}$ cardinal temperature data were included in niche co-specialisation analyses. * Listed on the IF/SF and Mycobank databases as *Phytophthora drechsleri* var. *cajani.* ** The European and Mediterranean Plant Protection Organization Global (EPPO) database notes *P. erythroseptica* var. *erythroseptica* as an alternative name of *P. erythroseptica* (https://gd.eppo.int/taxon/PHYTER) [accessed 6/3/2020]. *** Only *Ustilago nuda f.sp. hordei* was recorded in the Plantwise database for the species *U. nuda*. Hence, *U. nuda* in the Togashi dataset assigned hosts of *U. nuda f.sp. hordei* recorded in the Plantwise database.

**Species name in Plantwise database**	**Updated species name**
Acremonium strictum	*Sarocladium strictum*
Alternaria macrospora	*Alternaria brassicae*
Ascochyta gossypii	*Ascochyta gossypiicola*
Ascochyta pisi	*Didymella pisi*
Botryosphaeria obtusa	*Peyronellaea obtusa*
Botryosphaeria ribis	*Neofusicoccum ribis*
Cochliobolus heterostrophus	*Bipolaris maydis*
Cochliobolus miyabeanus	*Bipolaris oryzae*
Cochliobolus nodulosus	*Curvularia nodulosa*
Cochliobolus sativus	*Bipolaris sorokiniana*
Cochliobolus stenospilus	*Bipolaris stenospila*
Diaporthe phaseolorum	*Phomopsis phaseoli*
Diaporthe phaseolorum var. *caulivora*	*Phomopsis phaseoli*
Diaporthe phaseolorum var. *meridionalis*	*Phomopsis phaseoli*
Diaporthe phaseolorum var. *sojae*	*Phomopsis phaseoli*
Diaporthe vaccinii	*Phomopsis vaccinii*
Emericella nidulans	*Aspergillus nidulans*
Fusarium coeruleum	*Fusarium caeruleum*
Fusarium phaseoli	*Neocosmospora phaseoli*
Geotrichum candidum	*Dipodascus geotrichum*
Gibberella acuminata	*Fusarium acuminatum*
Gibberella avenacea	*Fusarium avenaceum*
Gibberella baccata	*Fusarium lateritium*
Gibberella fujikuroi	*Fusarium fujikuroi*
Gibberella fujikuroi var. *subglutinans*	*Fusarium fujikuroi*
Gibberella gordonii	*Fusarium lolii*
Gibberella intricans	*Fusarium gibbosum*
Gibberella pulicaris	*Fusarium roseum*
Gibberella tricincta	*Fusarium tricinctum*
Gibberella zeae	*Fusarium graminearum*
Glomerella cingulata	*Colletotrichum gloeosporioides*
Glomerella tucumanensis	*Colletotrichum falcatum*
Guignardia bidwellii	*Phyllosticta ampelicida*
Guignardia citricarpa	*Phyllosticta citricarpa*
Haematonectria haematococca	*Neocosmospora solani*
Helicobasidium brebissonii	*Helicobasidium purpureum*
Inonotus radiatus	*Xanthoporia radiata*
Khuskia oryzae	*Nigrospora oryzae*
Leptosphaeria coniothyrium	*Paraconiothyrium fuckelii*
Leptosphaeria sacchari	*Epicoccum sorghinum*
Leucostoma persoonii	*Cytospora leucostoma*
Magnaporthe grisea	*Pyricularia grisea*
Magnaporthe salvinii	*Nakataea oryzae*
Meria laricis	*Rhabdocline laricis*
Monographella nivalis	*Microdochium nivale*
Mycosphaerella fragariae	*Ramularia grevilleana*
Mycosphaerella graminicola	*Zymoseptoria tritici*
Mycosphaerella pinodes	*Didymella pinodes*
Nectria coccinea	*Fusarium lateritium*
Nematospora coryli	*Eremothecium coryli*
Neofabraea alba	*Neofabraea vagabunda*

Olpidium brassicae	*Olpidiaster brassicae*
Ophiostoma piceae	*Pesotum piceae*
Passalora fulva	*Fulvia fulva*
Penicillium aurantiogriseum var. *viridicatum*	*Penicillium aurantiogriseum*
Penicillium purpurogenum	*Talaromyces purpureogenus*
Penicillium rubrum	*Talaromyces ruber*
Peronospora hyoscyami f.sp. tabacina	*Peronospora hyoscyami*
Pestalotiopsis theae	*Pseudopestalotiopsis theae*
Phoma destructiva	*Boeremia exigua*
Phoma destructiva	*Remotididymella destructiva*
Phoma pinodella	*Didymella pinodella*
Phoma tracheiphila	*Plenodomus tracheiphilus*
*Phytophthora drechsleri f.sp. cajani**	*Phytophthora drechsleri*
Phytophthora erythroseptica var. *erythroseptica***	*Phytophthora erythroseptica*
Pilidiella diplodiella	*Coniella diplodiella*
Pleospora betae	*Pleospora bjoerlingii*
Pleospora herbarum	*Stemphylium vesicarium*
Porodaedalea pini	*Phellinus pini*
Pyrenochaeta terrestris	*Setophoma terrestris*
Pythium debaryanum	*Globisporangium debaryanum*
Pythium spinosum	*Globisporangium spinosum*
Pythium splendens	*Globisporangium splendens*
Pythium ultimum	*Globisporangium ultimum*
Pythium vexans	*Phytopythium vexans*
Setosphaeria monoceras	*Exserohilum monoceras*
Setosphaeria turcica	*Exserohilum turcicum*
Sphacelotheca reiliana	*Sporisorium reilianum*
Thanatephorus cucumeris	*Rhizoctonia solani*
Thielaviopsis basicola	*Berkeleyomyces basicola*
Tolyposporium ehrenbergii	*Anthracocystis ehrenbergii*
Uromyces trifolii	*Uromyces trifolii-repentis*
*Ustilago nuda f.sp. hordei****	*Ustilago nuda*
Valsa mali	*Cytospora mali*
Valsa sordida	*Cytospora chrysosperma*

Supplementary Table 12. Host names updated in the Plantwise database. See Methods for details of how hosts were renamed. Family Chenopodiaceae was absent from S.PhyloMaker. Rather than update this family name, we instead assumed Chenopodiaceae included *Allenrolfea*, *Aphanisma*, *Arthrocnemum*, *Atriplex*, *Axyris*, *Bassia*, *Beta*, *Camphorosma*, *Chenopodium*, *Corispermum*, *Cycloloma*, *Dysphania*, *Enchylaena*, *Endolepis*, *Exomis*, *Grayia*, *Halimione*, *Halocnemum*, *Halogeton*, *Krascheninnikovia*, *Maireana*, *Microtea*, *Monolepis*, *Nitrophila*, *Polycnemum*, *Proatriplex*, *Salicornia*, *Salsola*, *Sarcobatus*, *Sarcocornia*, *Spinacia*, *Suaeda, Suckleya,* and *Zuckia* genera (https://plants.usda.gov/classification.html) [accessed 16/5/2020]. For all hybrid (X) species, we assumed that species names matched between the Plantwise database and The Plant List, even if X was missing from species names recorded in the Plantwise database. * Only genus-level records were updated.

Host name in the Plantwise database	Corrected host name for phylogeny construction
Abies borisii-regis	*Abies* X *borisii-regis*
Acroptilon repens	*Rhaponticum repens*
Allium chinense	*Allium tuberosum*
Allium porrum	*Allium ampeloprasum*
Alnus viridis	*Alnus alnobetula*
Anthoxanthum puelii	*Anthoxanthum aristatum*
Aster ericoides	*Symphyotrichum ericoides*
Brassica chinensis	*Brassica rapa*
Brassica oleracea var. *botrytis*	*Brassica cretica*
Brassica oleracea var. *italica*	*Brassica cretica*
Brassica pekinensis	*Brassica rapa*
Capsicum frutescens	*Capsicum annuum*
Cassia obtusifolia	*Senna obtusifolia*
Centaurea cyanus	*Cyanus segetum*
Chamomilla recutita	*Matricaria chamomilla*
Chenopodium ambrosioides	*Dysphania ambrosioides*
Chrysanthemum frutescens	*Argyranthemum frutescens*
Cinnamomum zeylanicum	*Cinnamomum verum*
Cissus rhombifolia	*Cissus alata*
Citrus aurantium	*Citrus* X *aurantium*
Citrus bergamia	*Citrus limon*
Citrus deliciosa	*Citrus reticulata*
Citrus limonia	*Citrus limon*
Citrus macrophylla	*Citrus aurantiifolia*
Citrus unshiu	*Citrus reticulata*
*Coleus**	*Plectranthus**
Conyza canadensis	*Erigeron canadensis*
Coronilla varia	*Securigera varia*
Crambe abyssinica	*Crambe hispanica*
Crocosmia crocosmiiflora	*Crocosmia* X *crocosmiiflora*
Dioscorea batatas	*Dioscorea polystachya*
Dioscorea rotundata	*Dioscorea cayennensis*
*Dizygotheca**	*Schefflera**
Dracaena deremensis	*Dracaena fragrans*
Eucalyptus calophylla	*Corymbia calophylla*
Eucalyptus citriodora	*Corymbia citriodora*
Eutrema wasabi	*Eutrema japonicum*
Fragaria ananassa	*Fragaria* X *ananassa*
Garcinia mangostana	*Garcinia* X *mangostana*
Gleditsia caspica	*Gleditsia caspia*
Glycine soja	*Glycine max*
Hippophae rhamnoides	*Elaeagnus rhamnoides*
Kalanchoe pinnata	*Bryophyllum pinnatum*
Larix lubarskii	*Larix* X *lubarskii*
Lithocarpus densiflorus	*Notholithocarpus densiflorus*
Luffa aegyptiaca	*Luffa cylindrica*
Malus prunifolia	*Malus* X *prunifolia*
Medicago denticulata	*Medicago polymorpha*
Melilotus alba	*Melilotus albus*
Mentha piperita	*Mentha* X *piperita*
Michelia compressa	*Magnolia compressa*
Michelia doltsopa	*Magnolia doltsopa*
Musa paradisiaca	*Musa* X *paradisiaca*
Pascopyrum smithii	*Elymus smithii*

Pharbitis nil	*Ipomoea nil*
Pharbitis purpurea	*Ipomoea purpurea*
Photinia fraseri	*Photinia* X *fraseri*
Poncirus trifoliata	*Citrus trifoliata*
*Poncirus**	*Citrus**
Populus canadensis	*Populus* X *canadensis*
Populus maximowiczii	*Populus suaveolens*
Populus tomentosa	*Populus* X *tomentosa*
Quamoclit vulgaris	*Ipomoea quamoclit*
Raphanus sativus	*Raphanus raphanistrum*
Rhamnus frangula	*Frangula alnus*
Rhamnus purshiana	*Frangula purshiana*
Rheum hybridum	*Rheum* X *hybridum*
Rubus fruticosus	*Rubus plicatus*
Salix matsudana	*Salix babylonica*
Samanea saman	*Albizia saman*
Sesbania exaltata	*Sesbania herbacea*
Solanum gilo	*Solanum aethiopicum*
Solanum nigrum	*Solanum americanum*
Sorghum almum	*Sorghum* X *almum*
Sorghum dochna	*Sorghum bicolor*
Sorghum sudanense	*Sorghum* X *drummondii*
Spiraea bumalda	*Spiraea* X *bumalda*
Sterculia urens	*Firmiana simplex*
Tagetes patula	*Tagetes erecta*
Thymus citriodorus	*Thymus* X *citriodorus*
Triticum dicoccum	*Triticum dicoccon*
Ulmus procera	*Ulmus minor*
Vaccinium oxycoccus	*Vaccinium microcarpum*
Xanthocyparis nootkatensis	*Cupressus nootkatensis*
Zanthoxylum piperitum	*Zanthoxylum bungeanum*
Ziziphus mauritiana	*Ziziphus jujuba*

Supplementary Table 13. Influence of T_{max} reporting uncertainties on phylogenetic signal reported for *Phytophthora* species.

Phylogeny	Blomberg's *K*	*p*
Bayesian	0.574	0.001
Maximum likelihood	0.256	0.001
Maximum Parsimony	1.24	0.001

References

1. Yang, X., Tyler, B. M. & Hong, C. An expanded phylogeny for the genus Phytophthora. *IMA Fungus* **8**, 355–384 (2017).
2. Togashi, K. *Biological characters of plant pathogens: temperature relations*. (Meibundo, 1949).
3. Magarey, R. D., Sutton, T. B. & Thayer, C. L. A Simple Generic Infection Model for Foliar Fungal Plant Pathogens. *Phytopathology™* **95**, 92–100 (2005).
4. Martin, F. N., Abad, Z. G., Balci, Y. & Ivors, K. Identification and Detection of Phytophthora: Reviewing Our Progress, Identifying Our Needs. *Plant Disease* **96**, 1080–1103 (2012).
