# Supplementary material for: Geometry and evolution of the ecological niche in plant-associated microbes
Source: Nat Commun. 2020 Jun 11;11:2955. doi: 10.1038/s41467-020-16778-5 (PMC7289842; doi:10.1038/s41467-020-16778-5)
Supplement: Supplementary file 4 — Description of Additional Supplementary Files [file 41467_2020_16778_MOESM4_ESM.pdf]

Description of Additional Supplementary Files

File Name: Supplementary Data 1
Description: Species-biological process-cardinal temperature sample sizes for data presented in the Togahsi dataset.
